# Surface Fluorination of Magnesium Powder: Enhancing High-Temperature Oxidation Resistance

**DOI:** 10.3390/ma18235307

**Published:** 2025-11-25

**Authors:** Yu Wang, Jae-Ho Kim, Susumu Yonezawa

**Affiliations:** 1 Department of Materials Science and Engineering, Faculty of Engineering, University of Fukui, 3-9-1 Bunkyo, Fukui 910-8507, Japan; wyd23003@g.u-fukui.ac.jp; 2 Cooperative Research Center, University of Fukui, 3-9-1 Bunkyo, Fukui 910-8507, Japan; yonezawa@matse.u-fukui.ac.jp

**Keywords:** magnesium, surface fluorination, oxidation resistance, powder metallurgy

## Abstract

This study investigates the high-temperature oxidation mechanism of pure magnesium powder and the effect of surface fluorination on its oxidation resistance. The results showed that at high temperatures, pure Mg powder reacted with H_2_O and CO_2_ in air to form Mg(OH)_2_ and MgCO_3_, which decomposed at approximately 350 °C. Above 450 °C, the oxide film ruptured and catastrophic oxidation occurred. Surface fluorination with F_2_ gas generated a dense, uniform MgF_2_ protective film on the magnesium surface, significantly improving the ignition point and high-temperature oxidation resistance of Mg. Increasing the fluorination temperature increased the thickness and stability of the MgF_2_ layer, thereby further enhancing oxidation resistance. In particular, samples fluorinated at 200 °C showed oxidation growth limited to approximately 3%, even after heating at 500 °C for 8 h in air. Adjusting the surface fluorination conditions can create a protective MgF_2_ film to address high-temperature oxidation issues in magnesium powder metallurgy applications.

## 1. Introduction

Magnesium (Mg) has a low density, high specific strength and stiffness, low cutting resistance, strong impact-vibration resistance, and good recyclability, and is known to be the best green material of the 21st century [1]. Magnesium has received extensive attention as a lightweight material suitable for applications in autonomous vehicles and aerospace [2,3,4]. However, its low ignition point, poor high-temperature oxidation resistance, poor corrosion resistance, and other factors limit the application and development of magnesium. Song et al. and Yang et al. assessed the future developments of magnesium and mentioned that the future research direction should be the control of the microstructure in magnesium organization to reduce the process cost and obtain high-performance magnesium materials [1,5]. Powder metallurgy is a method for manufacturing metal materials from metal powders [6]. Compared to cast parts, materials prepared by powder metallurgy exhibit superior mechanical properties and corrosion resistance [7,8]. Furthermore, powder metallurgy products have smaller grain sizes than those prepared from extruded ingots [9]. Powder metallurgy is a low-cost, low-energy technique that produces high-performance products [10,11]. Akkas, Kumar, and Dvorský et al. used powder metallurgy to prepare Mg materials with significantly improved performance. They also summarized the problems associated with Mg powder during the production process [6,7,10]. Increasing the heating rate can significantly reduce the porosity of the powder [10]. The mechanical properties of the sintered products obtained under high-temperature conditions were even better [7]. However, the oxide layer of Mg thickened sharply, and as the temperature increased further, it hindered the sintering process [10]. An increase in temperature also increases the oxygen content in the tissue, coarsens the grains, and makes proper shaping difficult [7]. The oxide generated between the powders creates a porous structure, leading to the formation of microcracks that ultimately affect the mechanical properties of the sintered product [11]. Moreover, Mg is prone to combustion at high temperatures, which poses safety threats during production [12,13]. The low boiling point and high vapor pressure of Mg are the main reasons for Mg powder evaporation, as observed by Wei et al. [14]. Han et al. investigated the complex surface combustion of Mg and found that the ignition point was affected by the material itself as well as the external environment, and that Mg powder was more likely to burn than other materials. The authors pointed out that the ignition point of Mg and the growth of a high-temperature oxide film are closely linked [15]. To improve the sintering efficiency and reduce the risk of combustion, the oxidation resistance of Mg powder at high temperatures must be improved. The poor oxidation resistance of Mg at high temperatures is due to its high affinity for oxygen. The Pilling–Bedworth ratio (PBR; the ratio of the volume of the metal oxide after the reaction to the volume of the metal before the reaction) of magnesium oxide is usually less than 1. This indicates that the oxide layer generated on Mg is loose and porous, which cannot prevent Mg ions from continuing to diffuse and react with the oxygen in the air [16,17,18,19]. Increasing the density of the Mg surface layer can improve the oxidation resistance and ignition point of Mg [20]. To improve the oxidation resistance of Mg powders, various methods such as anodizing [21,22], chemical vapor deposition [23,24], and sol–gel coating [25,26] have been used. However, most studies have focused on corrosion, hydrophobicity, or antibacterial performance, with limited attention paid to high-temperature oxidation.

Surface fluorination of metals and alloys can be particularly effective and can significantly improve their corrosion resistance [27]. This may be due to the formation of a magnesium fluoride (MgF_2_) layer on the Mg surface. Because the PBR value of MgF_2_ is 1.29, the fluoride layer can be used as a protective film for Mg. Owing to its high thermodynamic stability and a decomposition temperature of approximately 2260 °C, the MgF_2_ layer remains intact and is not decomposed or degraded even under high-temperature oxidation conditions. To prepare the MgF_2_ layer on Mg, the SF_6_ gas has been used. However, alternatives are desirable owing to their high cost and associated environmental pollution [28]. Mirak and Toghyani et al. reported the surface treatment of magnesium-based materials using fluorination with HF [23,29,30]. A dense and smooth MgF_2_ protective film was generated on the surface of magnesium-based materials, significantly improving their properties. However, the fluorination treatment with HF was not uniform, and the dense protective layer was not formed locally, which led to the formation of magnesium oxide at high temperatures, hindering the sintering process [29,31]. Among the fluorinating agents, fluorine (F_2_) gas has high chemical activity and can be easily cleaved into fluorine radicals; therefore, direct fluorination treatment with fluorine gas is low-cost, simple, and effective [32]. The effectiveness of surface fluorination with F_2_ gas has been proven for both metallic and organic materials [33,34,35,36,37]. Powder metallurgy research on pure magnesium powder indicates that the powder reacts more with oxygen under high-temperature conditions owing to its larger surface area. In practical powder metallurgy applications of pure Mg, the extensive formation of MgO on the powder surface during high-temperature processing deteriorates the sinterability and degrades the properties of the final products. Therefore, alloying or surface treatment of the powder is typically performed prior to sintering to enhance the high-temperature oxidation resistance of the surface protective layer. However, studies on the surface fluorination of magnesium powders using F_2_ gas and systematic evaluations of their long-term high-temperature oxidation behavior are still lacking.

In this study, we summarize the reaction mechanism and surface structure of pure Mg powder during high-temperature oxidation. A safe and efficient direct fluorination scheme was designed using F_2_ gas for the surface fluorination of Mg samples. A dense and uniform MgF_2_ protective film was generated on the magnesium surface by adjusting the fluorination temperature during the process.

## 2. Materials and Methods

### 2.1. Oxidation Mechanism Testing of Pure Magnesium Powder

Pure Mg powder (KISHIDA CHEMICAL Co., Ltd., Osaka, Japan) was used in this study. Table 1 shows the elemental composition of the Mg powder, which was measured using energy-dispersive X-ray spectroscopy (EDS; EDX-7200; Shimadzu, Tokyo, Japan). Laser diffraction analysis (LDA; MT-3000 II; MICROTRAC, Osaka, Japan) was employed to determine the particle size distribution of the pure Mg powder using ethanol as the dispersion medium.

To check the oxidation resistance at high temperatures, 20 mg of each of the untreated pure Mg and fluorinated samples were placed in a thermogravimetric and differential thermal analysis (TG/DTA; TG/DTA 6300, Seiko Instruments Inc., Tokyo, Japan) apparatus. The temperature was increased from 25 to 550 °C in air at a rate of 30 °C/min, and data were collected every 0.5 s [15]. Subsequently, under the same experimental conditions, pure Mg powder was heated to different temperatures in the TG/DTA apparatus and maintained for different durations. Samples were prepared using a resistance furnace under the same conditions as those in the TG/DTA experiment, with an isothermal holding time of eight hours at different temperatures. All samples were investigated using elemental and morphological analyses via X-ray diffraction (XRD; XRD-6100, Shimadzu, Tokyo, Japan), X-ray photoelectron spectroscopy (XPS; JPS-9010, JEOL, Tokyo, Japan), and ultra-high-resolution field-emission scanning electron microscopy (FE-SEM; GeminiSEM560, Zeiss Ultra Plus, Jena, Germany).

### 2.2. Surface Fluorination of Pure Magnesium Powder

Before fluorination, Mg powder was placed in a Ni reactor (24 × 32 × 5 mm^3^) at 25 °C under vacuum (0.1 Pa) for 10 h to eliminate impurities from the system. The vacuum reactor was then filled with fluorine gas, and surface fluorination was performed under the conditions listed in Table 2. After fluorination, the reactor was purged with Ar.

### 2.3. Material Characterization

A reduction in the surface free energy can improve the driving force for sintering [38]. All fluorinated powder samples were subjected to high-temperature holding experiments using TG/DTA. To avoid changes in the surface composition when the samples were left in air for a long time, all samples were preserved by passing Ar gas (concentration: 99.9%; Uno Corp., Fukui, Japan) through them before and after the experiments were conducted. All the fluorinated powders, as well as the fluorinated powders after heat treatment, were investigated using elemental and morphological analyses via XRD, XPS, and FE-SEM.

## 3. Results and Discussion

### 3.1. Oxidation Mechanism of Pure Magnesium (Mg) Powder

#### 3.1.1. High-Temperature Oxidation of Raw Magnesium Powder

**Figure 1:**
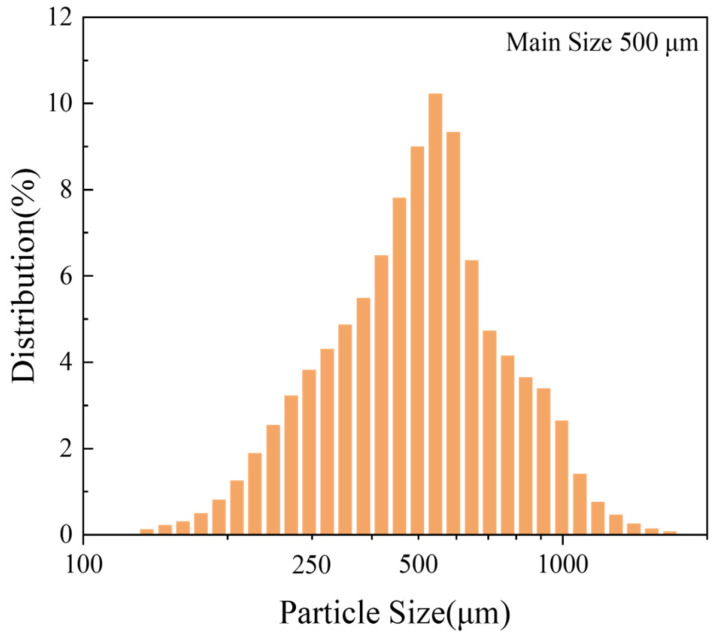
Statistical distribution of the particle sizes of pure Mg.

The particle size distribution of the pure Mg powder used in this study is shown in Figure 1. Finer powders have a higher surface area, and research has shown that finer powders have a lower ignition point [15], which can easily pose safety issues during their production. Therefore, for a comprehensive consideration of safety and cost, pure magnesium particles (main size of approximately 500 μm) were used as the sample in this experiment.

**Figure 2:**
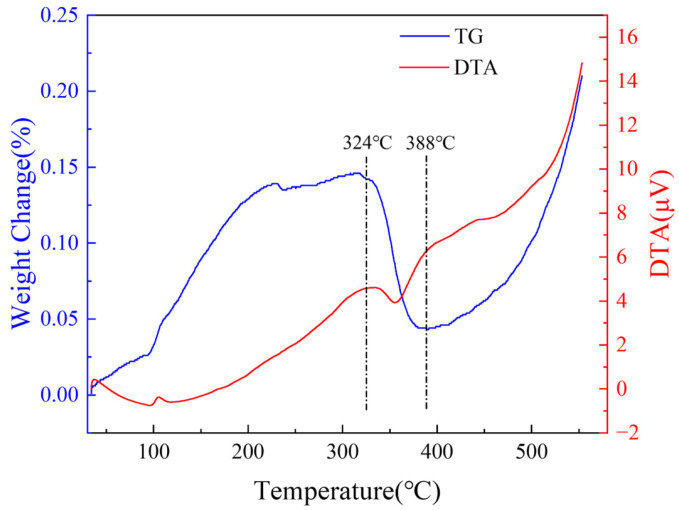
TG/DTA results of untreated samples heated from 25 to 550 °C.

**Figure 3:**
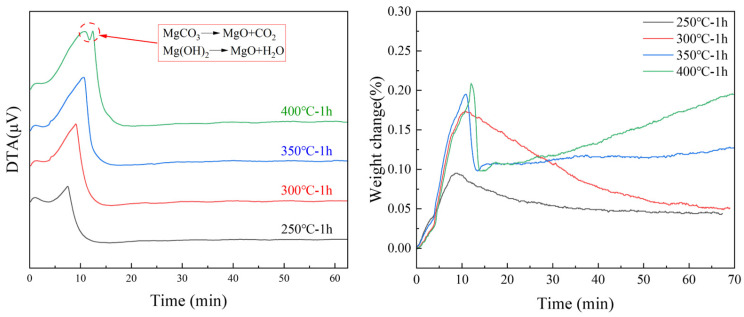
TG/DTA results of untreated samples heated from 25 °C to a set temperature for 1 h.

The TG results for the magnesium samples showed a significant mass drop in the early stage (324–388 °C) of heating, as shown in Figure 2. At this stage, the DTA detected an exothermic peak, which was accompanied by a heat absorption reaction. The decomposition temperatures of MgCO_3_ and Mg(OH)_2_ were above 350 °C. The DTA data indicated a significant heat absorption peak during the heating process at 400 °C, as shown in Figure 3. The mass drop shown in Figure 2 caused the decomposition of MgCO_3_ and Mg(OH)_2_. At lower temperatures, the mass appeared to decrease according to the TG results (Figure 3). The mass further decreased at lower holding temperatures. Because the oxidation reaction was exothermic and MgO had good thermal insulation, the actual interface temperature was higher than the heating temperature [39]. Therefore, it is likely that the surface reached its decomposition temperature. A phase with a very slow mass decrease rate was observed above 350 °C. However, this phase lasted only three minutes, after which the mass continued to increase.

#### 3.1.2. High-Temperature Isothermal Treatment

**Figure 4:**
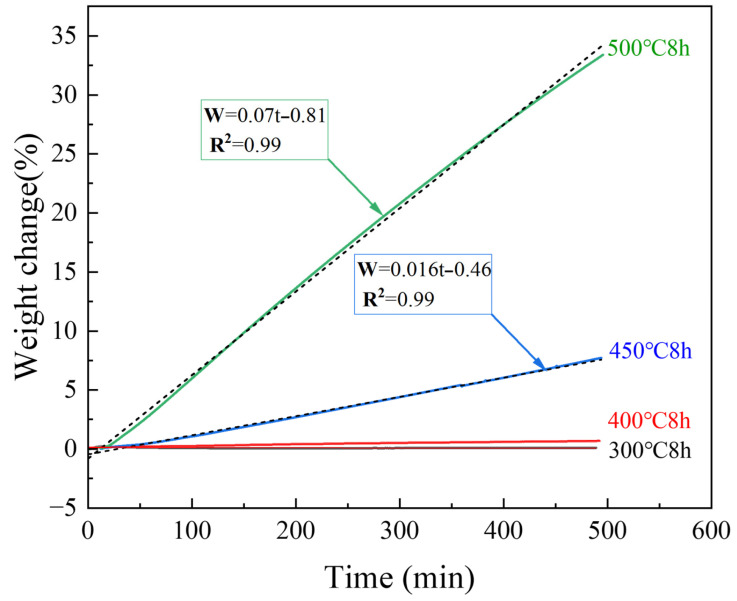
TG results of untreated samples at various temperatures.

**Figure 5:**
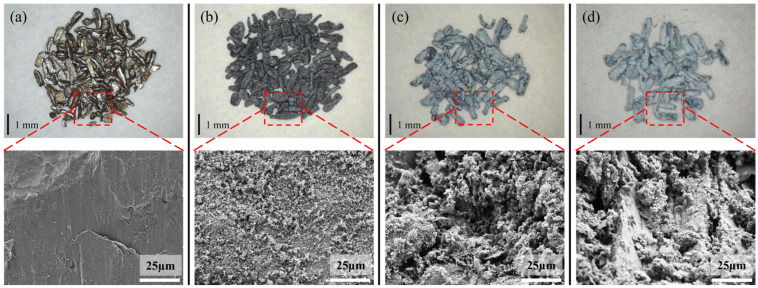
Microscope pictures and SEM images of the untreated samples after heating to various temperatures for 8 h. [(**a**) 300 °C, (**b**) 400 °C, (**c**) 450 °C, (**d**) 500 °C].

Figure 4 shows the TG results of untreated samples for 8 h isotherms at different temperatures. The TG results of the samples heated at 300 °C exhibited almost no change, and the surface still possessed a metallic luster and dense oxide layer, as shown by the microscope and SEM images presented in Figure 5a. The weight change (Figure 4) of the samples heated at 400 °C for 8 h was also almost negligible; however, the surface became black, and a large number of white magnesium oxide particles were produced on the Mg surface, as shown in the SEM images. Nevertheless, the oxide film retained some strength and did not break, as shown in Figure 5b. Figure 4 shows that as the temperature continued to increase, the mass growth at 450 and 500 °C was a straight line according to oxidation kinetics. The magnesium oxide film was not protective and could not prevent the combination of Mg ions and oxygen. As shown in Figure 5c,d, the surface became a white, unprotected, thick film of magnesium oxide. The SEM results showed that this layer was loose and porous, proving that the PBR of MgO was 0.81. The surface contained many cauliflower-like structures, indicating significant evaporation of Mg.

**Figure 6:**
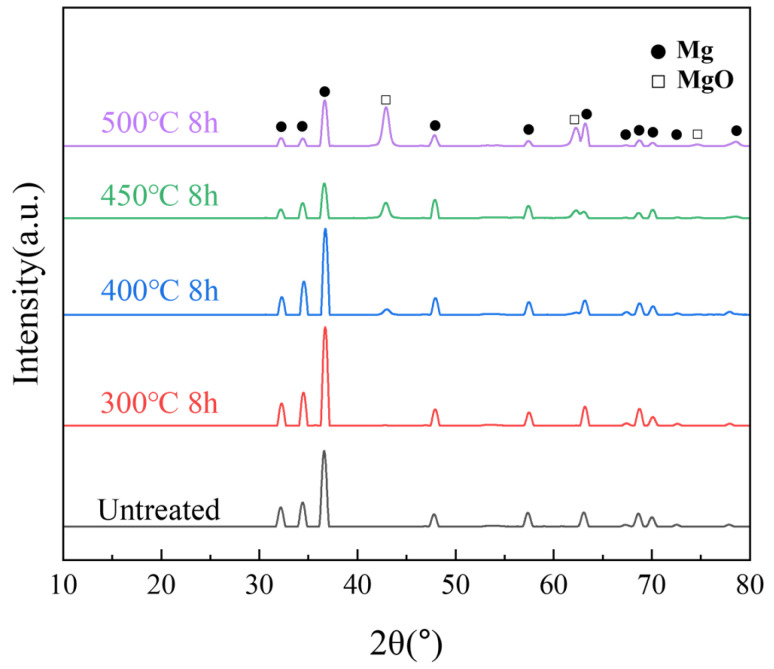
XRD patterns of untreated samples after different isothermal heat treatments.

XRD is commonly used to investigate the crystalline structure information of powders. The XRD results of the samples heated below 400 °C showed only the Mg peak, as shown in Figure 6. As the temperature increased, the internal grain size increased, and the intensity of the Mg peaks decreased. A new peak originating from MgO was detected near 44° at temperatures above 400 °C. The higher the temperature, the higher was the intensity of the MgO peak. The growth of MgO was significantly larger at 450 and 500 °C. A certain amount of MgO began to form inside the powder, indicating that high-temperature oxidation reactions occurred not only on the surface but also more severely within the interior of the particles.

**Figure 7:**
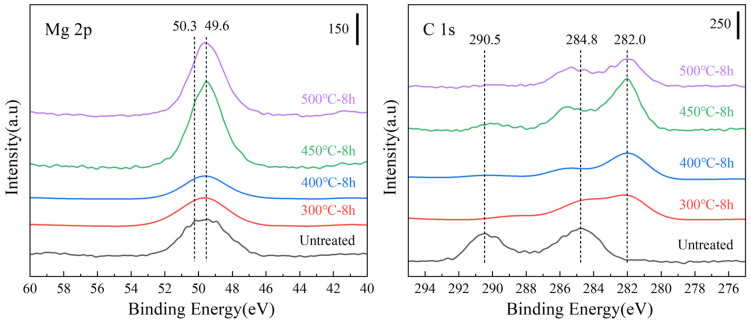
Mg 2p and C 1s X-ray photoelectron spectroscopy (XPS) spectra of the untreated powder after different isothermal heat treatments.

**Figure 8:**
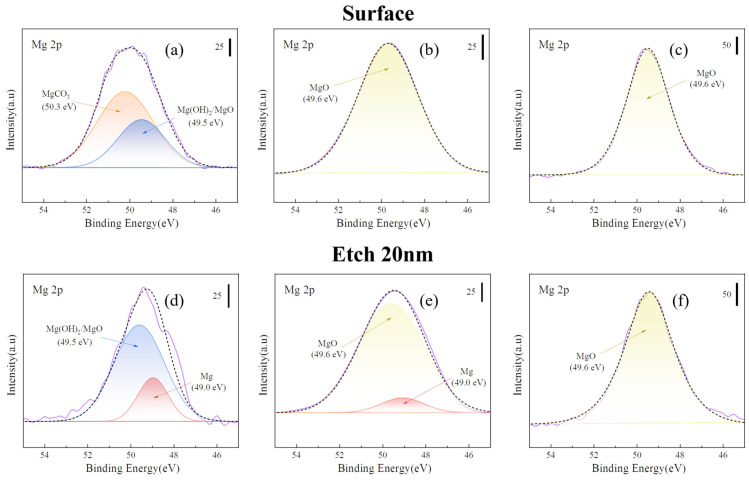
Peak separation of Mg 2p in Figure 7. [(**a**) untreated; (**b**) 300 °C-8 h; (**c**) 500 °C-8 h; (**d**) etching 20 nm of (**a**), (**e**) etching 20 nm of (**b**), and (**f**) etching 20 nm of (**c**)].

In the XPS (Mg 2p) results shown in Figure 7, the peaks of the samples heated at 300 and 400 °C for 8 h were more concentrated at the MgO position (49.6 eV) than that of the untreated sample. In the case of XPS (C 1s), the MgCO_3_ peak (290.5 eV) disappeared after heat treatment, indicating that the outermost layer of MgCO_3_ decomposed after heat treatment. The peak height of MgO (49.6 eV) increased for the samples heated at 450 °C and 500 °C for 8 h, indicating an increase in the MgO content on the surface of the films. The peak-splitting results (Figure 8) of the Mg 2p peaks of all the samples presented in Figure 7 indicate that the surface of the untreated sample (Figure 8a) was composed of a large amount of MgCO_3_. The MgCO_3_ on the surface disappeared completely after sputtering to a 20 nm thickness. After etching to 20 nm, the surface comprised Mg(OH)_2_, MgO, and a small amount of basal magnesium.

Figure 8b shows that the surface MgCO_3_ decomposed after heat treatment at 300 °C for 8 h. The internal Mg(OH)_2_ decomposed to magnesium oxide (MgO). However, the oxide film appeared to be very thin, and a small amount of the Mg substrate could be detected by sputtering to a depth of 20 nm. However, for the samples heated at 500 °C for 8 h, only the MgO peak was observed on the surface and inner 20 nm, as shown in Figure 8c. This indicates that the oxide layer generated at 500 °C was thick and dense.

### 3.2. Surface Fluorination of Magnesium Powder Under Various Conditions

**Figure 9:**
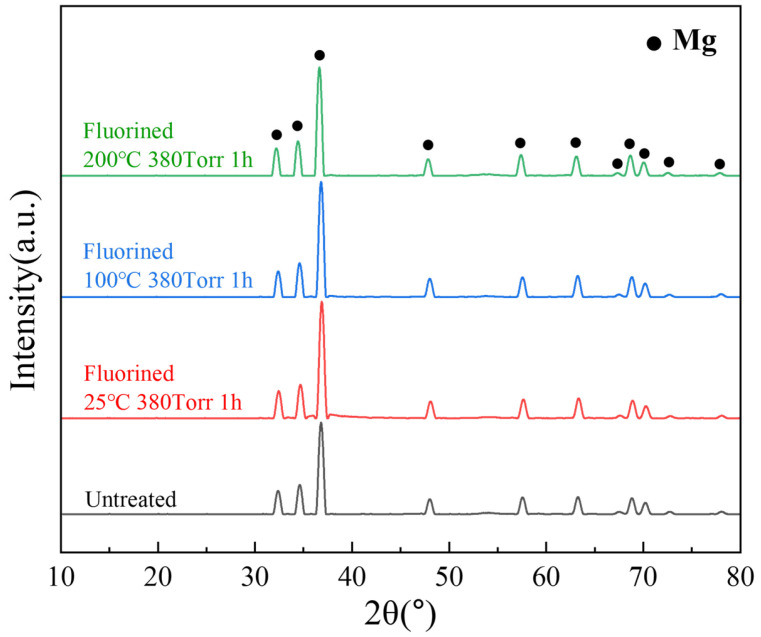
XRD patterns of untreated and fluorinated samples.

**Figure 10:**
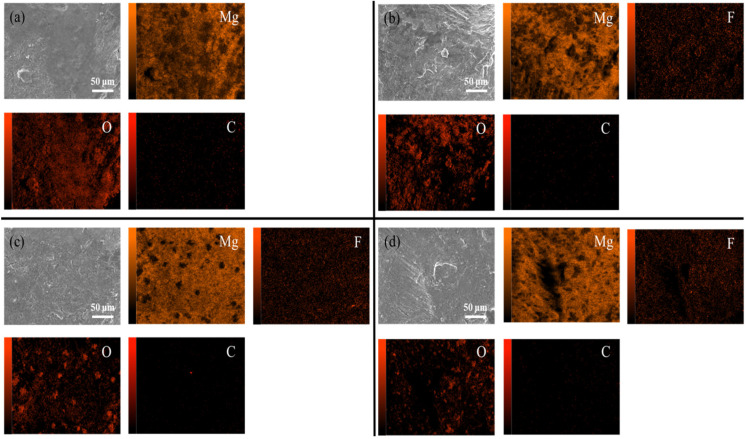
SEM/EDS images of untreated and fluorinated magnesium samples. [(**a**) Untreated, (**b**) F-25, (**c**) F-100 (**d**) F-200].

Figure 9 shows the internal XRD patterns of the untreated and fluorinated samples. Even after fluorination at 200 °C, no peak corresponding to MgF_2_ was observed in the XRD pattern. This implies that fluorination occurred on the surface layer and did not affect the internal crystal structure of the material. In the SEM and EDS analyses, compared with the untreated powder, the fluorinated powder exhibited a uniformly distributed fluorine signal on the surface, while the intensity of the oxygen signal decreased, indicating that the surface MgO was transformed into MgF_2_ (Figure 10).

**Figure 11:**
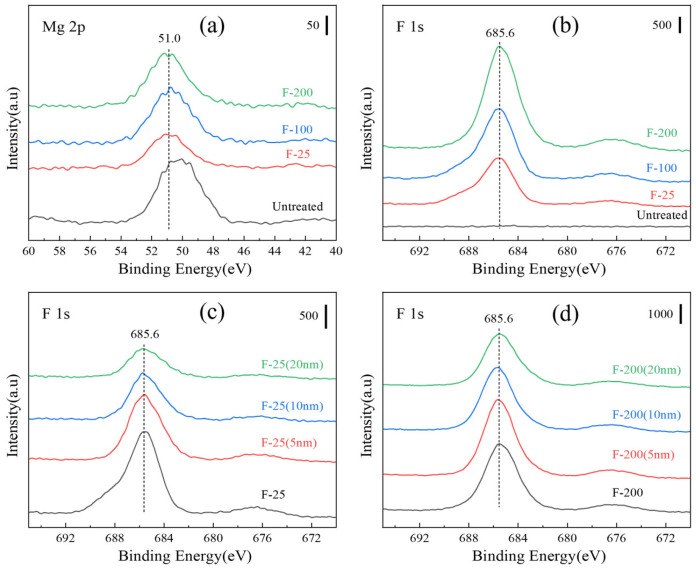
XPS spectra (**a**) various samples of Mg 2p and (**b**) F 1s; (**c**) depth spectra of F-25 samples and (**d**) F-200 samples.

Figure 11 shows the XPS spectra of the untreated and fluorinated Mg samples. All binding energies were calibrated to the C 1s peak of carbon at 284.8 eV binding energy. The Mg 2p peak of the fluorinated samples was shifted to 51.0 eV (MgF_2_), as shown in Figure 11a, and the F 1s peak was detected in all the fluorinated samples, as shown in Figure 11b. From Figure 11, it can be deduced that MgF_2_ was generated on the substrate surface. As shown in Figure 11c, the F content on the surface increased with increasing the fluorination temperature. The F content of the sample decreased after sputtering, indicating that MgF_2_ was formed mainly in the outermost layer, as shown in Figure 11d. Table 3 lists the elemental compositions of all the samples evaluated from the XPS results (Figure 11).

### 3.3. Effects of Surface Fluorination on the Oxidation Resistance of Magnesium

**Figure 12:**
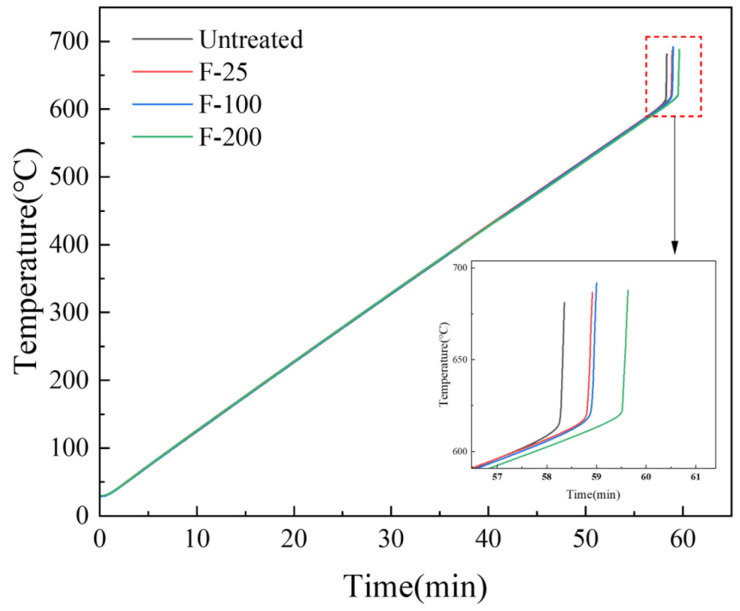
Ignition point test on untreated and fluorinated samples (heating range: 25–700 °C, heating rate: 10 °C/min).

Ignition point tests were performed on untreated and fluorinated samples at conditions—20–700 °C and a heating rate of 30 °C/min. The ignition point of pure magnesium is approximately 630 °C [40]. However, the ignition point of the pure Mg powder measured in this experiment was approximately 614.4 °C, as shown in Figure 12, which is due to the high surface area of the powder, making it more prone to oxidation reactions. The powder sample was more likely to burn because of its small size [15]. Figure 12 shows that the ignition points of the samples after the three fluorination treatments all increased, and the ignition point of F-200 increased by approximately 10 °C compared with that of the untreated sample.

**Figure 13:**
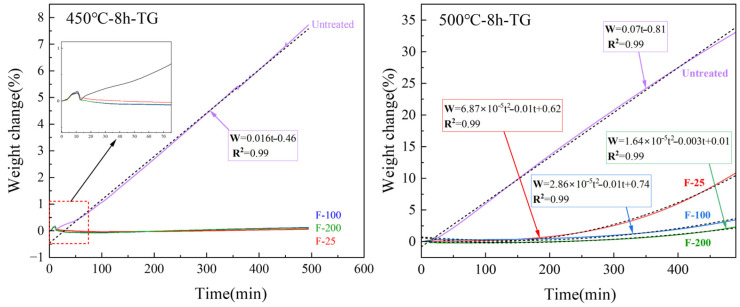
TG results of untreated and fluorinated samples heated at 450 and 500 °C for 8 h.

**Figure 14:**
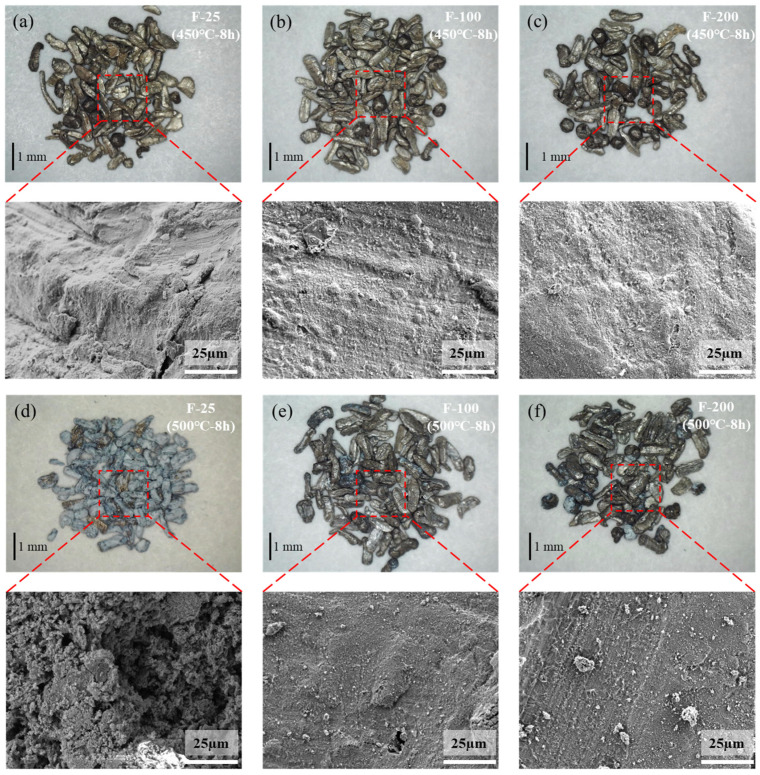
Microscope and SEM images of fluorinated samples after isothermal heating at 450 °C for 8 h [(**a**) F-25, (**b**) F-100, (**c**) F-200] and 500 °C for 8 h [(**d**) F-25, (**e**) F-100, (**f**) F-200].

Compared to the untreated samples, the fluorinated samples heated at 450 °C for 8 h in air presented almost no mass change, as indicated in Figure 13. The SEM images (Figure 14b,c) of the surfaces of the fluorinated samples after heating at 450 °C for 8 h in air are similar to those of the fluorinated sample surfaces before heating (Figure 10). White oxide films or cracks were not observed, as observed on the surface of the untreated sample (Figure 14a). This indicates that MgF_2_ can help protect the surface of Mg from oxidation at temperatures below 450 °C. However, upon increasing the temperature to 500 °C in air, the mass change with time curve of the fluorinated samples can be fitted as a quadratic equation based on the oxidation kinetics. This indicates that MgF_2_ on the surface has a protective effect against the continuous accumulation of Mg vapor. As the reaction time increased beyond 200 min, MgF_2_ on the surface continued to break down, and the oxidation rate changed from slow to fast, resulting in the continued growth of the oxide film. Consequently, the oxidation weight gain curve of the F-25 samples after 8 h was almost similar to that of the untreated sample, as shown in Figure 13. In the case of the F-25 samples (Figure 14d) subjected to heating at 500 °C for 8 h, the microscope image shows that a white oxide film was generated on the surface of the Al substrate. The oxide film was sparse and porous in the SEM images, and its surface morphology was similar to that of the untreated samples, as shown in Figure 5c,d. This indicates that the MgF_2_ layer on the F-25 samples was insufficient to protect against oxidation at temperatures beyond 500 °C in air; the weight change (%) of the F-100 and F-200 samples after 8 h was limited to 3.14 and 2.12, respectively. As shown in Figure 14e,f, although the surface started to become dull, it still appeared as a dense protective layer in the SEM images.

**Figure 15:**
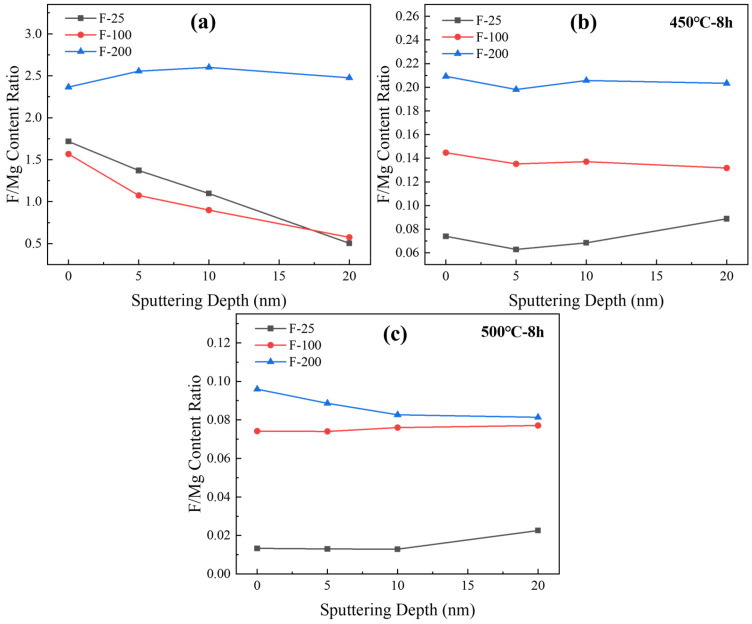
The ratio of elemental fluorine and magnesium content with sputtering depth of various samples. [(**a**) fluorinated powder before heat treatment, (**b**) fluorinated pow-der after 450 °C for 8 h, (**c**) fluorinated powder after 500 °C for 8 h].

The ratio of fluorine to magnesium effectively reflects the fluorine content on the sample surface. Figure 15a shows the ratios of fluorine and magnesium contents at different depths for the three groups of samples after fluoridation. The MgF_2_ content at different depths increased with the temperature. Figure 15b,c show the analysis of the elemental content on the surface of the samples after high-temperature heat treatment, and the results are consistent with Figure 15a, in which the MgF_2_ content increases with an increase in the temperature of the fluoridation treatment of the samples. Combined with the weight change shown in Figure 13, MgF_2_ improved the high-temperature antioxidant performance of pure Mg.

**Figure 16:**
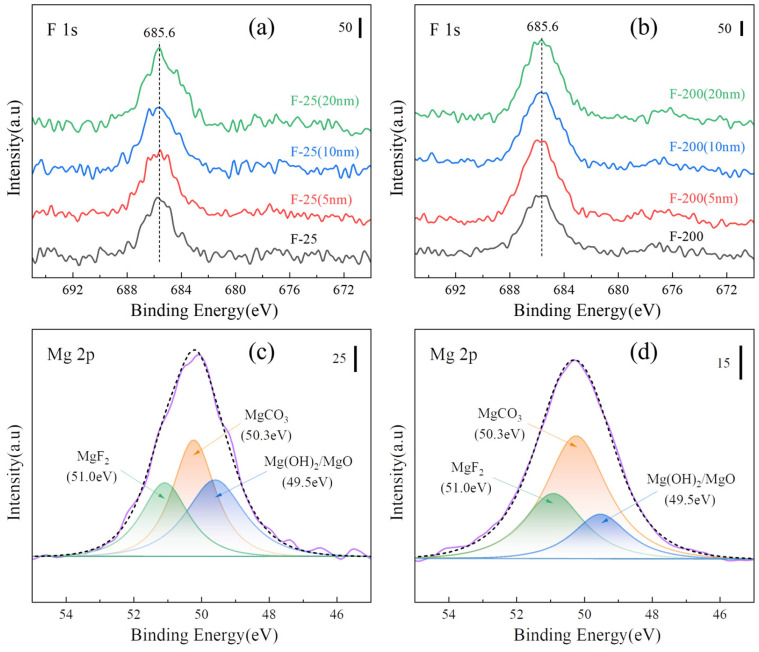
XPS spectra of various samples after isothermal heating at 450 °C for 8 h. [(**a**) F 1s of the F-25 surface and etching, (**b**) F 1s of the F-200 surface and etching, (**c**) peak separa-tion of Mg 2p on the F-25 surface, (**d**) peak separation of Mg 2p on F-200 surface].

**Figure 17:**
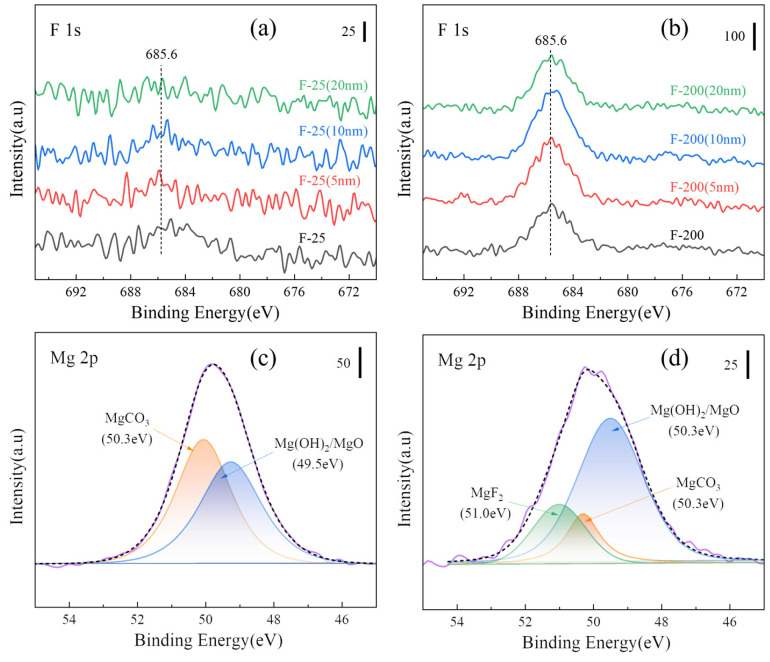
XPS spectra of various samples after isothermal heating at 500 °C for 8 h. [(**a**) F 1s of F-25 surface and etching, (**b**) F 1s of F-200 surface and etching, (**c**) peak separation of Mg 2p on F-25 surface, (**d**) peak separation of Mg 2p on F-200 surface].

Figure 16a,b show the peaks of XPS F 1s on the surface and after sputtering for F-25 and F-25 after heat treatment at 450 °C for 8h. The spectral peaks of F 1s can be detected both on the surface and inside 20 nm, and the surface is composed of MgF_2_, MgCO_3_, Mg (OH)_2_, and MgO by splitting the peaks on the surface, as shown in Figure 16c,d. The spectral peak of F 1s was not detectable from the surface to the internal 20 nm in Figure 17a, and the spectral peak of F 1s can be detected in Figure 17b. Figure 17c,d shows the peak separation of the surface, respectively. The F-25 surface had no MgF_2_, whereas F-200 still consisted of MgF_2_, indicating that F-200 has superior fluoride protection performance compared to F-25.

## 4. Conclusions

**Figure 18:**
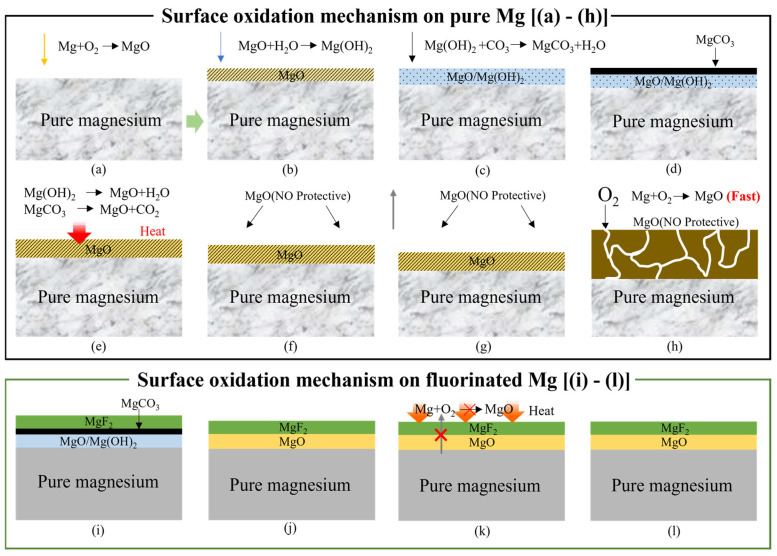
Surface oxidation mechanism of (**a**–**h**) untreated and (**i**–**l**) fluorinated magnesium samples according to different heating temperature. [(**a**–**d**): <350 °C, (**e**,**f**): 350–400 °C, (**g**,**h**): >450 °C, (**i**): <350 °C, (**j**–**l**): 350–500 °C].

Magnesium powder readily reacts with H_2_O and CO_2_ in air, forming Mg(OH)_2_ and MgCO_3_ on the outer surface, as shown in Figure 18a–d. Upon heating to approximately 350 °C, both Mg(OH)_2_ and MgCO_3_ decompose to MgO (Figure 18e). Below 400 °C, a relatively compact MgO layer forms on the surface, providing limited protection due to the slow oxidation kinetics in this temperature range. However, above 450 °C, the MgO film undergoes rupture and spallation, leading to the formation of a loose and non-protective white oxide scale (Figure 18f–h), which results in rapid and catastrophic mass gain during oxidation.

In contrast, fluorinated Mg powder using F_2_ gas produces a chemically bonded and dense MgF_2_ layer on the surface, which significantly enhances its high-temperature oxidation resistance (Figure 18i). Increasing the fluorination temperature increases both the thickness and stability of the MgF_2_ layer, thereby improving its long-term protective performance. Notably, the MgF_2_ layer formed under the F-200 condition effectively limited the oxidation mass gain to below ~3% after isothermal heating at 500 °C for 8 h in air.

These results demonstrate that controlled F_2_-based surface fluorination enables the formation of a uniform and adherent MgF_2_ protective film, effectively solving the long-standing problem of severe high-temperature oxidation in magnesium powder metallurgy.

## Figures and Tables

**Table 1:** Elemental composition (wt. %) of pure magnesium powder.

Mg	Ca	Mn	Cu
99.884	0.084	0.024	0.008

**Table 2:** Sample names and fluorination conditions.

Sample Name	Temperature (°C)	Time (h)	F_2_ Pressure (Torr)
untreated	-	-	-
F-25	25	1	380
F-100	100	1	380
F-200	200	1	380

**Table 3:** Elemental composition of samples evaluated from XPS results (Figure 11).

Samples	Elemental Contents (at %)		MgF_x_
	Mg	F	
untreated	23.79	-	MgF_0.00_
F-25	22.60	42.38	MgF_1.88_
F-100	25.60	50.12	MgF_1.95_
F-200	23.62	48.79	MgF_2.06_

## Data Availability

The original contributions presented in this study are included in the article. Further inquiries can be directed to the corresponding author.
